# Effectiveness of Non-Animal Chondroitin Sulfate Supplementation in the Treatment of Moderate Knee Osteoarthritis in a Group of Overweight Subjects: A Randomized, Double-Blind, Placebo-Controlled Pilot Study

**DOI:** 10.3390/nu11092027

**Published:** 2019-08-29

**Authors:** Mariangela Rondanelli, Valentina Braschi, Clara Gasparri, Mara Nichetti, Milena Anna Faliva, Gabriella Peroni, Maurizio Naso, Giancarlo Iannello, Daniele Spadaccini, Niccolò Miraglia, Pietro Putignano, Tariq A. Alalwan, Simone Perna

**Affiliations:** 1 IRCCS Mondino Foundation, 27100 Pavia, Italy; 2 Department of Public Health, Experimental and Forensic Medicine, Unit of Human and Clinical Nutrition, University of Pavia, 27100 Pavia, Italy; 3 Endocrinology and Nutrition Unit, Azienda di Servizialla Persona ‘‘Istituto Santa Margherita’’, University of Pavia, 27100 Pavia, Italy; 4 General Management, Azienda di Servizialla Persona Istituto Santa Margherita, 27100 Pavia, Italy; 5 Clinical & Pre-clinical Development, Gnosis SpA, 20121 Milan, Italy; 6 SP Diabetic Outpatient Clinic, ASST Monza, 20900 Monza, Italy; 7 Department of Biology, College of Science, University of Bahrain, Sakhir Campus P. O. Box 32038, Bahrain

**Keywords:** non-animal chondroitin sulfate, knee osteoarthritis, pain, inflammation, overweight, obesity

## Abstract

Osteoarthritis (OA) is the most common form of arthritis in the world and is characterized by pain, various disabilities and loss of quality of life. Chondroitin sulfate (CS) is recommended as first-line therapy. CS of non-animal origin is of great interest for safety and sustainability reasons. This study aims to investigate the anti-inflammatory effects, anti-pain and ability-enhancement of a short-term supplementation with non-animal CS in overweight subjects with OA. In a randomized, double-blind, placebo-controlled pilot study, 60 overweight adults with symptomatic OA were allocated to consume 600 mg of non-animal CS (*n* = 30) or a placebo (*n* = 30) daily for 12 consecutive weeks. The assessment of knee-pain, quality of life, related inflammation markers and body composition was performed at 0, 4 and 12 weeks. The Tegner Lysholm Knee Scoring (TLKS) scale of the experimental group showed a statistically significant increase (+10.64 points; confidence interval (95% confidence interval (CI) 5.57; 15.70; *p* < 0.01), while the Western Ontario and McMaster Universities Osteoarthritis Index (WOMAC) score decreased (−12.24 points; CI 95% −16.01; −8.38; *p* < 0.01). The results also showed a decrease in the C-reactive protein (CRP) level (−0.14 mg/dL, CI 95% −0.26; −0.04; *p* < 0.01) and erythrocyte sedimentation rate (ESR) level (−5.01 mm/h, CI 95% −9.18; −0.84, *p* < 0.01) as well as the visual analogue scale (VAS) score in both knees. In conclusion, this pilot study demonstrates the effectiveness of non-animal CS supplementation in overweight subjects with knee OA in improving knee function, pain and inflammation markers.

## 1. Introduction

Knee osteoarthritis (OA) is predicted to become the fourth leading cause of disability worldwide by 2020 [1] and is estimated to affect more than 40 million people in Europe [2] and 4 million people in Italy [3]. OA has multifactorial etiology, and obesity is one of the most important risk factors for knee OA [4,5,6], together with a sedentary lifestyle. Knee OA is associated with joint stiffness, pain, and impairment in joint functions and its prevalence increases with age.

Disease progression is associated with cartilage degradation, joint stiffness, joint space narrowing, pain and motility impairment. OA is further related to local and systemic inflammation. Inflammatory mediators are produced by articular tissues and are involved in disease pathogenesis.

Fat mass is a risk factor for cartilage degradation and for every 1 kg increase in total body fat there is an increased risk of cartilage defects. Mainly for this reason, overweight or obese people have a major risk to develop a cartilage defect specially at the knee joint [7]. Moreover, the weight load on the knees is not the only risk factor for joints damage, because adipocytes with the production of many pro-inflammatory cytokines contribute to the synovial inflammation and bone matrix remodeling [8,9].

Regarding therapy of OA, the European Society for Clinical and Economic Aspects of Osteoporosis and Osteoarthritis (ESCEO) published a treatment algorithm to manage patients with knee OA [10]. In Step 1 of the treatment, it is recommended to initiate therapy with chronic symptomatic slow-acting drugs for osteoarthritis (SYSADOAs). Among SYSADOAs, evidence is greatest for the effect of chondroitin sulfate (CS).

The European League against Rheumatism (EULAR) recommendations for the management of knee OA are dated in 2003 and stated that the effect of CS supplementation may have been exaggerated by publication bias [11]. Also, the most recent evidence-based recommendations by the Osteoarthritis Research Society International (OARSI) consider the recommendation of CS for disease modification as “not appropriate” and for symptom relief as “uncertain” [12].

The United Kingdom’s National Institute for Health and Care Excellence (NICE) has recommended that these products should not be used, mainly for economic reasons, while the American College of Rheumatology (ACR) recommended CS under certain conditions, but concluded that evidence supporting the use of CS for pain relief was mostly low level [13].

Nevertheless, different studies have demonstrated its anti-inflammatory potential which is important for reducing pain, edema, inflammation, and allowing matrix regeneration in cartilage damage [14,15,16]. Furthermore, some studies have also demonstrated a CS effect against obesity-related inflammation and atherogenesis (although it was reported for mice and cultured coronary endothelial cells, the data needs to be validated in human trials) since it counteracts the pro-inflammatory activation of monocytes and endothelial cells caused by tumor necrosis factor-α (TNF-α) and macrophage infiltration in adipose tissue [17,18].

The cytokines level and other inflammatory indicators, including C-reactive protein (CRP) and erythrocyte sedimentation rate (ESR), are often altered in OA, although usually are into the upper level of the normal range. Joint discomfort and motility impairment may also lead the affected individuals to higher levels of sedentariness, thus causing an increase in body weight and affecting the body mass index (BMI). Obesity is often related to OA and is one of the primary risk factors for developing both knee and hand osteoarthritis [19]. In this context, in the present study, parameters related to body composition have been monitored as markers of general health improvement linked to a recovery in motility and physical activity.

The poor quality of many animal-derived CS preparations and the intrinsic safety issues related to animal-derived products, due to the presence of several contaminants (immunogenic keratan sulfate, hyaluronic acid, unknown proteins, nucleic acids), raises an interest in biotechnological, non-animal-derived CS. Although several patents on CS processes have appeared, none of these products have demonstrated its biological activity.

This is the first reported study investigating the effectiveness of a non-animal CS on pain and motility in patients with knee OA. Microbial-derived CS (Mythocondro^®^) has previously been tested in vitro and in animal effectiveness models showing a greater efficacy in reducing pro-inflammatory cytokines plasma levels and several specific arthritic parameters in rats [20]. Commonly used CS in clinical trials and therapy is of animal origin.

A recent Cochrane Review of 2016 demonstrated that chondroitin was associated with statistically significant lower odds of serious adverse events compared with placebo, and adverse events were reported in a limited fashion, with some studies providing data and others not [21].

Although the studies on the safety of animal CS claim a positive feedback, the drawbacks are related to poor reproducibility and the lack of an appropriate control of the extracted material, in addition to the potential safety concerns associated with the possible presence of transmissible infective agents, such as viruses and bacteria [22]. Contamination by other polysaccharides such as immunogenic keratan sulfate and hyaluronic acid or nucleic acids is also possible, as well as the presence of unknown proteins and other macromolecules [23,24]. Moreover, the consumption of animal CS may be avoided for religious or cultural reasons. Finally, the position and percentage of sulfate groups can also vary in relation to specific animal sources, thus giving a different absorption profile [22,25].

For all these reasons, non-animal CS is watched with interest. Alternately to the extraction of CS from animal tissues, a fermentation-based process has been developed. After a fermentation step from the capsular polysaccharides K4 of *Escherichia coli*, the process is followed by two chemical steps of defructosilation and selective sulfation. Non-animal CS has already demonstrated a comparable or even better activity to animal CS for both clinical and biochemical parameters in an experimental model of adjuvant-induced arthritis in rats [20]. As compared to animal CS, non-animal CS has also shown a better bioavailability when administrated in healthy volunteers in two pharmacokinetic clinical studies [22,26].

Considering this background, this clinical study investigated the anti-inflammatory and anti-pain potential of a short-term supplementation with non-animal CS in overweight subjects with OA. Moreover, we also assessed body composition by dual energy X-ray absorptiometry (DXA) in order to evaluate the change in fat free mass related.

## 2. Materials and Methods

### 2.1. Population

Using a monocentric, prospective, randomized, double-blind, placebo-controlled pilot study design, 60 subjects (male and female) with OA were recruited through the metabolic rehabilitation division of the Santa Margherita Hospital (Azienda di Servizialla Persona, Pavia, Department of Public Health, University of Pavia, Pavia, Italy) from late January 2017 to the end of June 2018.

The study was approved by the ethics committee of the Department of Internal Medicine and Medical Therapy of the University of Pavia. Participants received information on all aspects of the study and were provided with a written consent form to be signed prior to participation in the study. Data were gathered before participation in the study. Each potential subject completed a medical test screening, including demographic information, medical history, current medications and supplements, vital signs, blood tests, urine tests, and a 12-lead electrocardiogram. Anyone with evidence of heart, kidney or liver disease, or any other disease that might influence the results of the study was excluded. Trial registration: NCT03731793.

### 2.2. Inclusion/Exclusion Criteria

The inclusion criteria comprised Caucasian sedentary subjects, males or females, with the following characteristics: overweight individuals (BMI between 25 and 30 kg/m^2^), aged 55 years and over, experiencing mobility impairment, joint discomfort or established moderate knee OA (classification 1–3 according to the Kellgren and Lawrence scoring system for classification of knee OA) and a pain intensity value of 40–70 mm, measured using the visual analogue scale (VAS). The exclusion criteria comprised clinically significant abnormal physical findings which could interfere with the objectives of the study, clinically significant abnormal laboratory values indicative of physical illness and affected by pathologies, ascertained or presumptive hypersensitivity to the active ingredient tested (i.e., CS), excessive smokers and people with abuse of alcohol, significant history of diseases to lung, liver, heart and kidney that could interfere with the aim of the study, subjects affected or treated for rheumatoid arthritis, and consumption of nutraceuticals or food supplements containing CS for 2 weeks before the start of the study. Recruited participants were instructed to avoid nonsteroidal anti-inflammatory-drugs (NSAIDs) and high-CS-containing foods (animal cartilages, bones, or derivatives such as gelatin) which are known for their anti-inflammatory and/or analgesic effects. In addition, volunteers were instructed not to consume nutraceuticals or food supplements containing CS during the study.

### 2.3. Description of the Intervention

Eligible men and women were randomly assigned to one of the two groups: the experimental group (*n* = 30) or the placebo group (*n* = 30). The experimental group received 600 mg of Mythocondro^®^ daily for 12 weeks. Mythocondro^®^ was provided by Gnosis by Lesaffre (Desio, Italy), a business unit of the Lesaffre Group (Marcq-en-Baroeul, France). Table 1 shows the experimental design for this trial.

Test product: Mythocondro^®^ is an ichthyic-like CS produced by chemical sulfation of a non-sulfated chondroitin backbone obtained by thermo-acid hydrolysis of the capsular polysaccharide naturally produced by a specific strain of *E. coli* (O5:K4:H4 strain U1-41(ATCC23502)).

Ichthyic-like is related to, or characteristic of, fish but not from an animal source.

The strain is not genetically modified and the final content of endotoxins is monitored to guarantee the safety of the final product. The final content of endotoxins is determined by an Limulus amebocyte lysate (LAL) test. The non-animal CS produced according to the process (Mythocondro^®^) is a highly purified, standardized and well-characterized chemical substance. Mythocondro^®^ is characterized by the presence of sulfate groups in defined positions, by a constant charge density and a defined molecular mass range. Mythocondro^®^ is substantially monosulfated, mainly on the 6-carbon position (C6S), possessing few or no sulfation on the 4-carbon position (C4S), and being very similar to ichthyic natural CS. Mythocondro^®^ is further characterized by the absence of trisulfated and polysulfated disaccharides and having a lower molecular weight (1000–5000 daltons) than CS extracted from animal tissues. Its charge density ranges from 1.0 to 1.25. Mythocondro^®^ sulfation pattern and its charge density confer to this polysaccharide a shark-like composition. The batch-to-batch homogeneity is warranted by analytical controls aimed to deliver batches according to a strictly defined specification sheet. Mythocondro^®^ is also characterized by an extremely low protein contamination (≤0.5%) batch-to-batch controlled, with an evident advantage in avoiding allergic reactions in consumers.

The experimental product was presented in the form of a 1.02 g tablet, containing 600 mg of Mythocondro^®^ and excipients such as microcrystalline cellulose, mannitol, stearic acid, polyvinylpyrrolidone (PVP), silicon dioxide, sodium croscarmellose and magnesium stearate. Each participant was asked to consume one tablet a day for 12 weeks to be swallowed with a glass of water. The placebo product consisted of tablets (1.02 g) of similar flavor and appearance as the experimental product. Placebo tablets contained microcrystalline cellulose, mannitol, stearic acid, PVP, silicon dioxide, sodium croscarmellose and magnesium stearate but lacked the active ingredient. Each participant consumed one placebo tablet per day for 12 weeks with the same modality as the experimental product.

### 2.4. Assessment of Experimental Product Tolerance

Non-animal CS tolerance was established by relying on the absence of side effects, i.e., gastrointestinal symptoms such as nausea and diarrhea. Participants were assessed for any side effects of supplements intake on a daily basis by telephone conversation with a registered dietician. None of the participants refused to take the supplement, and no side effects were reported.

### 2.5. Assessment of the Effectiveness of the Experimental Product

One of the main endpoints of the study was the evaluation of the analgesic potential of the tested product performed at the end of the supplementation period (12 weeks) by using the VAS scale and the Western Ontario and McMaster Universities Arthritis (WOMAC) index and the recovery of knee functions and ability using the Tegner Lysholm Knee Scoring (TLKS) scale.

The endpoints include the evaluation of the systemic anti-inflammatory potential of the tested product determined by evaluating CRP level and ESR in blood, the effect of the tested product on the improvement of quality of life after 12 weeks of supplementation using the ShortForm36 (SF-36) health survey, and the effect of the tested product on the body composition at week 4.

Eligible subjects were invited to the study site for three visits during the 12-week study period, which included a baseline visit (Time-0), follow-up visit (week 4), and end-of-study visit (week 12).

During each visit, knee pain and ability were evaluated using the WOMAC index and the TLKS scale, respectively. Furthermore, health-related quality of life was evaluated using the SF-36 questionnaire and inflammation markers were analyzed in plasma for CRP and whole blood for ESR. Body composition was measured by DXA, while pain intensity was measured every day both at motion and at rest on the VAS scale and treatment compliance was tracked every day together with VAS evaluation.

### 2.6. Pain and Knee Function Assessment

#### 2.6.1. Assessment of knee function by WOMAC Index

The WOMAC pain scale consists of five questions that assess pain while walking on a flat surface, going up or down stairs, in bed at night, sitting or lying, and standing upright. The responses are recorded on a five-point Likert scale, with a higher score representing a greater level of pain. This scale is validated and reliable in hip and knee OA populations [27]. The WOMAC measures five items for pain (score range 0–20), two for stiffness (score range 0–8), and 17 for functional limitation (score range 0–68). Physical functioning questions cover everyday activities such as stair use, standing up from a sitting or lying position, standing, bending, walking, getting in and out of a car, shopping, putting on or taking off socks, lying in bed, getting in or out of a bath, sitting, and heavy and light household duties.

#### 2.6.2. Assessment of knee symptoms and function by the TLKS Scale

Each patient completed a self-report questionnaire TLKS, related to knee symptoms and function. It includes the following eight items: limp, support, locking, instability, pain, swelling, stair climbing, and squatting. Each possible response to the items gives an arbitrary score on an increasing scale ranging from 0 to 100 with a higher score indicative of better ability [28].

#### 2.6.3. Visual Analogue Scale of Pain

VAS measures a characteristic or attitude ranging across a continuum of values and cannot be directly measured. For example, the amount of pain that a patient feels ranges across a continuum from none to an extreme amount of pain. The VAS score is determined by measuring in millimeters from the left hand end of the line to the point that the patient marks [29].

### 2.7. Assessment of Quality of Life

The SF-36 Bodily Pain Scale (SF-36 BPS) is one of eight subscales of the medical outcomes study SF-36 questionnaire, a generic measure of health status designed for use in population surveys. The Medical Outcomes Study (MOS) 36-item Short Form survey subscale assesses body pain as a dimension of health status [30].

### 2.8. Inflammation Biochemical Parameters

CRP level was measured by particle-enhanced immunone phelometry on a Behring Nephelometer analyzer using the relevant kit (Dade Behring, Marburg, Germany) while ESR was analyzed by capillary photometry method using Test-1 (Alifax, Padova, Italy). Blood samples were taken from each patient in their fasting state at baseline at 8.00 a.m.

### 2.9. Assessment of Body Composition

Body composition was measured at baseline and at week 12 by DXA, using a Lunar Prodigy DEXA (GE Medical Systems, Waukesha, WI, USA). Fat mass, android fat and visceral fat data were derived from DXA using the DXA Prodigy enCORE software (version 17; GE Healthcare, Waukesha, WI, USA). Visceral adipose tissue (VAT) volume was estimated using a constant correction factor (0.94 g/cm^3^). The software automatically places a quadrilateral box which represents the android region, outlined by the iliac crest and with a superior height equivalent to 20% of the distance from the top of the iliac crest to the base of the skull [31,32].

### 2.10. Run-In, Randomization and Masking

On completion of the baseline assessment, participants were provided with a 12-week supply of experimental or placebo study capsules (blinded to content) and the 60-day VAS. All participants were randomly assigned (1:1) to take, once daily, two capsules each of: non-animal CS or matching placebo.

### 2.11. Sample Size

Following the previous study by Cohen et al. in 2004 [33], a statistical power calculation performed prior to the trial determined that to detect a difference of 2 cm in VAS pain reduction between the placebo and active treatment groups with 80% power required at least 25 patients in each group (based on a 2-tailed, 2-sample t-test with 5% significance level and assuming the 2 groups had an equal standard deviation of 2.5 cm).

### 2.12. Statistical Analysis

Statistical analyses were performed using SPSS 21.0 (SPSS, Inc., Chicago, IL, USA). The sample size was not calculated as this was a pilot study. Mean and standard deviation (SD) for all variables were given as descriptive statistics.

Continuous variables were calculated between groups by means and SDs, including the endpoints, with Student’s two-sample t-test. Chi-square analysis and Fisher’s test were used for calculating differences for categorical variables, such as gender distribution between the experimental and the placebo group. The endpoints of this randomized trial were continuous variables, the analysis of covariance (ANCOVA) with pre-treatment measurements as a covariate (age) was used to compare the two groups as “mean change” pre-post treatment from week 0 to week 12. The general linear model (GLM) univariate analysis of covariance procedure provides regression analysis and analysis of variance for one dependent variable by one or more factors and/or variables. Using this GLM procedure, it is possible to test null hypotheses about the effects of other variables (for example, age) on the means of a single dependent variable. To estimate the intra-group effects of pre-post interventions (non-animal CS and placebo) on free fat mass (FFM), fat mass (FM), VAT, and all variables of SF-36, TLKS and WOMAC scale and inflammation markers (CRP and ESR), an analysis of covariance was performed using as a covariate the baseline values of age (age is a strong determinant). These age-adjusted differences (and their 95% confidence intervals, CI) between the experimental and the placebo group were estimated. The effects for each group across time (three different instances) were separately analyzed using ANCOVA for repeated measures. Repeated measures which assessed the interaction by time for type of intervention has been observed for the TLKS and WOMAC scales and inflammation markers (CRP and ESR). In addition, ANCOVA was used to assess between-group differences for all variables (group B versus group A) (and their 95% CI) using baseline age value (for the corresponding time) as a covariate. Differences for VAS scale values for each day and between groups were assessed comparing the mean value between groups with independent means. The level of statistical significance was set at *p* < 0.05.

## 3. Results

### 3.1. Characteristics at Baseline

As described in Table 1, a total of 60 subjects completed the study. No dropouts were experienced during the trial and no side effects were reported at any moment.

Descriptive statistics of the sample at baseline are presented in Table 2. There were no significant differences at baseline for any of the variables.

Table 3 shows the within-group mean changes from baseline to week 12. In addition, the same table shows the treatment effect between groups.

### 3.2. Pain and Motility

Within-group mean changes from baseline (the beginning to the end of the supplementation period) are shown in Table 3. Concerning the endpoints related to ability and pain, the increase in the TLKS score was significant in the group receiving non-animal CS +0.42 * points (6.24; 12.96). Also, the WOMAC score decreased (with a lower score representing an improvement of pain level over time) within the non-animal CS group −8.70 * points (−11.25; −6.14) and it was statistically significant compared to the placebo group. Between groups analysis showed that concerning the endpoints of ability, the increase in the TLKS score was significant in the group receiving non-animal CS when compared to the placebo group (+10.64 pts; CI 95%: 5.57; 15.70; *p* < 0.001). In particular, TLKS showed an increase (+13.71%) in the experimental group receiving non-animal CS and a decrease (−1.57%) in the placebo group (group A).The decrease in WOMAC scores over time for the non-animal CS group was statistically significant compared to the placebo group (−12.24 pts; CI 95%: −16.01; −8.38; *p* < 0.001).

### 3.3. Inflammation

As far as ESR is concerned, a significant reduction of −4.41 * (mg/dL) (−7.14; −1.69) was observed in the non-animal CS compared to the placebo group. Similarly, CRP showed a significant reduction of −0.08 * (mm/h) (CI 95%: −0.16; −0.01) in the non-animal CS group compared to the placebo group. Regarding the ESR, the between-groups analysis showed that the non-animal CS group had a significant reduction of −5.01 mm/h (CI 95%: −9.18; −0.84; *p* < 0.01) when compared to the placebo group. Pre-post treatment mean change was significant only in the experimental group (*p* < 0.05). Similarly, CRP showed a significant reduction of −0.14 mg/dL (CI 95%: −0.26; −0.04; *p* < 0.01) between the non-animal CS and the placebo group.

### 3.4. Body Composition

FFM improved significantly in the non-animal CS group +538.53 g (CI 95%, 52.16; 1024.90) (*p* < 0.05), while no significant variation was observed in the placebo group.

### 3.5. Quality of Life

No significant changes of the SF-36 evaluations were observed in the placebo group while in the non-animal CS group, the pain score increased by 5.99 points and 8.81 points, respectively (both *p* < 0.05). The between-groups difference for SF-36: Physical pain score was nearly significant (*p* = 0.053) at the end of the supplementation period (12 weeks).

### 3.6. Side Effects

No significant side effects were observed in the experimental group.

**Figure 1:**
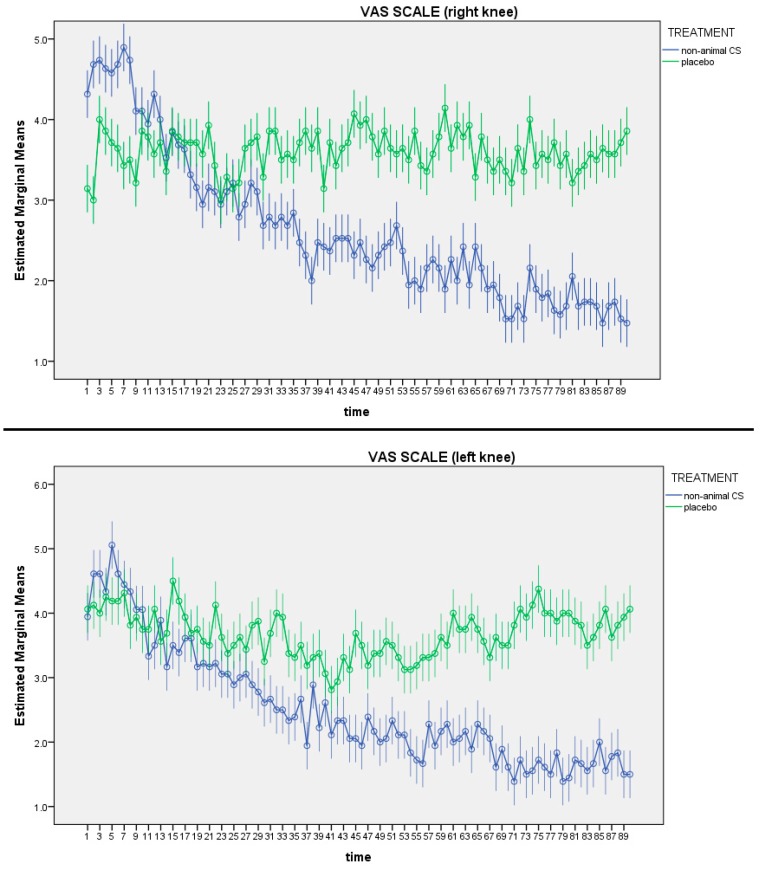
Visual analogue scale (VAS) of left and right knee. In both knees the VAS differences between groups are statistically significant over time (*p* < 0.05).

Figure 1 shows the differences in VAS values for each day and between groups were assessed comparing the mean value between groups with independent means. As shown in Figure 1, the VAS reported an improvement of pain in the non-animal CS group for both left and right knees (*p* = 0.001).

## 4. Discussion

In the present randomized, double-blind, placebo-controlled pilot study, the effectiveness of non-animal CS has been demonstrated in humans for the first time by supplementing 30 individuals affected by moderate knee OA with Mythocondro^®^ for 12 weeks versus a placebo.

The main finding of our study was the significant increase in the TLKS, representing an improvement in articular function and the decrease in WOMAC score, representing a decrease in the level of pain.

Regarding the short time effects of non-animal CS, only after four weeks the differences in pain (VAS scale) between the two groups were significant for both right and left legs and this gap increased over time.

These results are comparable to those obtained with animal CS. Regarding the benefits of CS, a recent meta-analysis by Honvo et al. [34] that represented a total of 3791 participants, 1886 of whom received oral CS, and 1905 were randomized to placebo, showed that CS provides moderate pain benefit in OA patients and has a large effect on function in knee OA, however with large inconsistency [34].

One of the possible explanations could be that pharmaceutical-grade CS preparations have a high degree of standardized purity, contrary to the non-pharmaceutical-grade preparations [35].

For this reason, the ESCEO have recommended only the patented pharmaceutical-grade preparations of crystalline glucosamine sulfate and CS to ensure clinical benefit for patients with OA [36].

Contrary to our results, a recent Cochrane review demonstrated that animal CS (alone or in combination with glucosamine) was better than placebo in improving pain in short-term studies including participants with osteoarthritis [21]. In this review, Singh et al. [21] compared forty-three randomized controlled trials of two weeks’ duration or longer in a meta-analysis comprising 4962 participants treated with chondroitin, alone or added with glucosamine, and 4148 participants given placebo or another control (NSAIDs). Most of these trials were in knee OA, with a few in hip and hand OA. WOMAC, VAS and Lequesne’s index were the major outcomes considered as well as the SF-36 questionnaire for the quality of life assessment. The review reports a reduction in knee pain (WOMAC). An improvement in the quality of life, knee function and disability has also been reported.

In the present study, a significant reduction of CRP plasma level and ESR was observed in the treated group over time as compared to the placebo group, indicating a clinically relevant activity.

Reduction of CRP and ESR anyway is statistically significant but moderately low, in addition our cohort of subjects being in the normal range at baseline, it could not be interpreted as an anti-inflammatory effect of non-animal CS. The explanation discussed is only an orientation of our data, with potential applications in subjects with CRP and ESR not in a normal range [37].

In accordance with our data, the recent systematic review and meta-analysis by Jin et al. highlights that in particular, a low-grade systemic inflammation may play a greater role in symptoms rather than radiographic changes in OA [38].

Recently, CRP reduction was also observed in adjuvant arthritic rats treated with Mythocondro^®^ by Bauerovaet al. [20]. The anti-inflammatory activity of Mythocondro^®^ is also in accordance with the described anti-inflammatory effect of CS at the synovial membrane and chondrocytes levels [39], where CS demonstrated the ability of counteracting interleukin 1 (IL1)-induced nuclear translocation of nuclear factor kB (NF-kB) in synoviocytes and chondrocytes.

Regarding body composition assessed by DXA, in the present study, FFM showed a significant improvement in the non-animal CS receiving group. This is likely related to the increase of daily activities due to the analgesic effects exerted by CS.

These findings have not been previously reported in the literature. Also the VAT decrease, although not reaching a formal statistical significance, maybe associated to the FFM increase itself or correlated with the reduction of inflammatory markers (CRP and ESR) found in the treated group, since VAT accumulation is a source of pro-inflammatory cytokines and is associated to a CRP increase [40].

Concerning the quality of life assessment, the SF-36 evaluations showed significance in the physical pain and physical activity sections when considering the intra-group variations. These results obtained with non-animal CS are in agreement with those reported with animal CS in The Multicenter Osteoarthritis intervention trial with SYSADOA (MOVES), but we have to highlight that a fixed combination of CS with glucosamine was used in the MOVES trial and not CS alone, as done in the present study.

The MOVES trial found that a fixed-dose combination of CS plus glucosamine has comparable efficacy to Celecoxib in reducing pain. Similar improvements were seen for VAS and for the pain/discomfort dimension of EuroQoL-5D [41]. On the other hand, Clegg et al. used the SF-36 to assess the health-related quality of life improvement in a subgroup of patients with moderate-to-severe knee pain and demonstrated that glucosamine and CS alone or in combination may be effective in order to treat knee OA [42].

In our study, we chose to evaluate overweight subjects because they are particularly at risk for developing knee OA. Osteoarthritic patients treated with high doses of CS have a lower incidence of coronary heart disease [17].

Recent studies in mice and cultured coronary endothelial cells have reported that CS treatment could lead to the formation of atheromatous plaque. CS also interferes with the TNF-α-induced secretion of IL-6 and IL-8 in monocytes and macrophages. These two locally secreted cytokines are directly related to some adipokines that are intimately linked to the development of atherosclerosis, as well as being a risk factor for cardiovascular disease in initially healthy populations [42]. Nevertheless, no meta-analysis has been carried out to establish this risk factor [17].

The present study was a pilot trial that was carried out on a moderate number of subjects over a relatively short period of time. The main limitation of the study is the high variability for markers of inflammation, that could affect the statistical significance in the difference between the groups.

In addition, we included general inflammation markers (but easy to assess in clinical practice). Furthermore, we did not assess the lack of cartilage (i.e., collagen type II, CTX-II) and synovium metabolism (i.e., hyaluronic acid) and lack of physical activity measurements to support the hypothesis of decrease related to FFM.

Probably due to the low number of subjects involved, body composition parameters did not reach a statistical significance. Despite all of these shortcomings, the results of this pilot study are very encouraging and the effectiveness of Mythocondro^®^ for the management of pain in OA should be confirmed in a larger number of patients for a longer period of treatment. Nonetheless, it is noteworthy that the beneficial effects of Mythocondro^®^ were present after a short-term treatment and at a lower dose than usual, with an improvement of the major OA-related parameters comparable to those reported for animal CS preparations [21].

Nevertheless, a specific follow up (from month to month) after the treatment has not been evaluated. We reported that the treatment discontinuation brought the patients in a pretreatment clinical symptomatology.

## 5. Conclusions

This pilot study, involving a moderate number of subjects, demonstrated for the first time in literature the effectiveness in improving knee function, pain and inflammation markers of a dose of 600 mg/day of non-animal CS supplementation in overweight people with knee OA. It is noteworthy that many of the beneficial effects were present even after four weeks of treatment. It is evident that there are advantages to the use of non-animal CS as a dietary supplement when considering potential side effect issues. Further studies evaluating the activity of both short-term and long-term supplementation with non-animal CS are necessary to confirm our preliminary findings.

## Figures and Tables

**Table 1:** Experimental design of the trial.

Treatment	Duration	OA Assessment Times
Placebo (30 subjects)	12 weeks	Pretreatment (T0), 4 week (T1), 12 week (T2)
Non animal CS (30 subjects)	12 weeks	Pretreatment (T0), 4 week (T1), 12 week (T2)

Abbreviations: OA, osteoarthritis; CS, chondroitin sulfate.

**Table 2:** Descriptive statistics of the sample at baseline.

Variables	PLACEBO (Mean; SD)	Non Animal CS (Mean; SD)	TOTAL SAMPLE (Mean; SD)	*P* Value between Groups at Baseline
**Demographic Characteristics**				
Gender (F: Female/M: Male)	30 (0F/10M)	30 (18F/12M)	60 (38F/22M)	0.568
Age (years)	62.77 (8.23)	62.53 (9.25)	62.64 (8.73)	0.924
Height (m)	1.62 (0.12)	1.59 (0.10)	1.60 (0.11)	0.321
Weight (kg)	74.52 (20.34)	71.76 (16.62)	72.98 (18.20)	0.609
**Body composition**				
BMI (kg/m^2^)	27.56 (3.38)	27.88 (3.60)	27.74 (3.47)	0.743
FFM (g)	41,977.77 (9000.14)	42,777.42 (8123.84)	42,425.58 (8440.48)	0.746
FM (g)	30,377.13 (12,528.26)	26,641.00 (12,845.20)	28,284.90 (12,715.93)	0.300
VAT	1185.50 (997.38)	1087.11 (725.64)	1131.28 (850.22)	0.701
**Knee pain and function**				
TLKS (0–100) pts	68.31 (12.00)	64.17 (14.64)	66.00 (13.57)	0.278
WOMAC (0–96) pts	42.93 (14.35)	46.22 (15.73)	44.77 (15.07)	0.445
**Quality of life**				
SF-36: Physical Activity	67.04 (25.66)	58.75 (16.97)	62.40 (21.41)	0.201
SF-36: Physical Role Limitations	59.77 (39.26)	54.46 (44.12)	56.80 (41.72)	0.655
SF-36: Physical Pain	49.00 (12.99)	46.32 (16.34)	47.50 (14.88)	0.552
SF-36: General Health	55.59 (15.17)	55.17 (16.22)	55.36 (15.60)	0.927
SF-36: Vitality	50.22 (13.75)	53.75 (16.47)	52.20 (15.29)	0.404
SF-36: Social Activities	71.90 (19.24)	70.28 (18.99)	71.00 (18.92)	0.767
SF-36: Emotional Role Limitations	77.18 (37.69)	71.32 (39.31)	73.90 (38.32)	0.595
SF-36: Mental Health	58.00 (17.94)	64.14 (16.24)	60.97 (17.27)	0.235
**Inflammation**				
ESR (mm/h)	22.31 (20.01)	16.42 (6.80)	19.02 (14.35)	0.198
CRP (mg/dL)	0.22 (0.26)	0.18 (0.24)	0.20 (0.25)	0.603

The values in the table are expressed as mean value (standard deviation, SD). Abbreviations: CS, chondroitin sulfate; BMI, body mass index; FFM, free fat mass; FM, fat mass; VAT, visceral adipose tissue; TLKS, Tegner Lysholm Knee Score; WOMAC, Western Ontario and McMaster Universities Osteoarthritis Index; ESR, erythrocyte sedimentation rate; CRP, C-reactive protein.

**Table 3:** Within-group mean changes from baseline (from day 0 to the end of the supplementation) for clinical markers. * in bold: value with *p* < 0.05.

Variables	PLACEBO Intra-Group Δ Change and CI 95%	Non animal CS Intra-Group Δ Change and CI 95%	Treatment Effect between Groups (Non-Animal CS Minus Placebo) Δ Change and CI 95%	*p*-Value between Groups
**Body composition outcomes**				
Weight (kg)	−2.29 (−8.61; 4.02)	−3.75 (−9.37; 1.88)	−1.45 (−9.91; 7.01)	0.507
BMI (kg/m^2^)	0.07 (−0.22; 0.36)	−0.18 (−0.45; 0.08)	−0.253 (−0.65; 0.14)	0.140
FFM (g)	81.92 (−466.79; 630.62)	538.53 * (52.16; 1024.90)	456.61 (−276.66; 1189.89)	0.150
FM (g)	−68.70 (−810.54; 673.14)	−719.38 (−1376.94; −61.81)	−650.67 (−1642.05; 340.70)	0.134
VAT (g)	−223.41(−391.89; −54.94)	−164.16 (−335.52; +7.21)	59.26 (−181.39; 299.91)	0.240
**Knee pain and function**				
TLKS (score)	−1.04 (−4.83; 7,75)	9.60 * (6.24; 12.96)	**10.64 (5.57; 15.70)**	***p* < 0.001**
WOMAC (score)	3.54 (0.65; 6.42)	−8.70 * (−11.25; −6.14)	**−12.24 (−16.01; −8.38)**	***p* < 0.001**
**Quality of life**				
SF-36: Physical Activity (score)	0.05 (−4.54; 5.93)	5.99 * (1.43; 10.55)	5.294 (−1.65; 12.23)	0.091
SF-36: Physical Role Limitations (score)	−4.19 (−19.47; 11.09)	6.14 (−7.16; 19.45)	−10.33 (−30.54; 9.92)	0.215
SF-36: Physical Pain (score)	−0.58 (−7.75; +6.60)	8.81 * (2.57; 15.06)	9.39 (−0.12; 18.91)	0.053
SF-36: General Health (score)	0.06 (−6.11; 6.24)	−0.32 (−5.70; 5.05)	−0.39 (−8.58; +7.80)	0.641
SF-36: Vitality (score)	0,10 (−5.25; 8.84)	0.53 (−5.60; +6.67)	−1.26 (−10.60; 8.08)	0.546
SF-36: Social Activities (score)	−8.09 (−17.14; 0.94)	−0.37 (−8.24; 7.50)	7.72 (−4.26; 19.71)	0.139
SF-36: Emotional Role limitations (score)	−6.25 (−19.97; 7.46)	1.30 (−13.23; 10.75)	4.96 (−13.23; 23.51)	0.406
SF-36: Mental Health (score)	−2.00 (−8.90; 4.90)	−4.07 (−9.84; 1.71)	−2.06 (−11.70; 6.94)	0.449
**Inflammation markers**				
ESR (mm/h)	0.04 (−2.56; 3.75)	−4.41 * (−7.14; −1.69)	**−5.01 (−9.18; −0.84)**	***p* < 0.01**
CRP (mg/dL)	0.21 (−2.56; 3.75)	−0.08 * (−0.16; −0.01)	**−0.14 (−0.26; −0.04)**	***p* < 0.01**

Abbreviations: CS, chondroitin sulfate; BMI, body mass index; FFM, free fat mass; FM, fat mas; VAT, visceral adipose tissue; TLKS, Tegner Lysholm Knee Score; WOMAC, Western Ontario and McMaster Universities Osteoarthritis Index; ESR, erythrocyte sedimentation rate; CRP, C-reactive protein.
